# Emerging Limb Rehabilitation Therapy After Post-stroke Motor Recovery

**DOI:** 10.3389/fnagi.2022.863379

**Published:** 2022-03-23

**Authors:** Fei Xiong, Xin Liao, Jie Xiao, Xin Bai, Jiaqi Huang, Bi Zhang, Fang Li, Pengfei Li

**Affiliations:** ^1^Department of Operation Room, The First People’s Hospital of Jiande, Hangzhou, China; ^2^Department of Orthopedics, The First People’s Hospital of Jiande, Hangzhou, China

**Keywords:** stroke, rehabilitation, limb motor function, mirror therapy, robot-assist therapy, motor imagery, music therapy, visual reality

## Abstract

Stroke, including hemorrhagic and ischemic stroke, refers to the blood supply disorder in the local brain tissue for various reasons (aneurysm, occlusion, etc.). It leads to regional brain circulation imbalance, neurological complications, limb motor dysfunction, aphasia, and depression. As the second-leading cause of death worldwide, stroke poses a significant threat to human life characterized by high mortality, disability, and recurrence. Therefore, the clinician has to care about the symptoms of stroke patients in the acute stage and formulate an effective postoperative rehabilitation plan to facilitate the recovery in patients. We summarize a novel application and update of the rehabilitation therapy in limb motor rehabilitation of stroke patients to provide a potential future stroke rehabilitation strategy.

## Introduction

Stroke is an acute cerebrovascular disease with high morbidity, mortality, and disability. It is the second leading cause of death worldwide, accounting for 11.6% of deaths. According to the Global Burden of Disease report, an estimated 12.2 million strokes are there worldwide, resulting in 143 million disability-adjusted life years (DALYs) and 6.55 million deaths (GBD 2019 Stroke Collaborators, 2021). China has the highest number of stroke cases globally. The number of patients belonging to the low-income and youth groups is rapidly increasing, with significant gender and regional differences. According to the WHO, in 2019, stroke was the leading cause of death and DALYs in China (World Health Organization [WHO], 2020). Stroke results in lasting sensory, cognitive and visual impairment, impaired limb motor function, and eventually reduce various bodily functions (Katzan et al., 2018a,b). Motor dysfunction is the most common complication of stroke, followed by hemiplegia in about 80% of patients. Half of these symptoms will accompany patients for life and seriously affect their day-to-day activities (Kim et al., 2020). Studies have shown that hemiplegia is the leading cause of long-term disability in stroke patients from the United States, Japan, and France [(Leys et al., 2008; Ovbiagele and Nguyen-Huynh, 2011; Iso, 2021)]. The fatality rate is significantly lower than before with the progress and development of stroke treatment. However, 80% of the survivors have severe sequelae, and the disability rate is about 75% (Langhorne et al., 2018). Effective rehabilitation training can alleviate functional disability, restore the motor function in hemiplegic limbs, and accelerate the rehabilitation process in post-stroke patients (Laver et al., 2020). At present, patient rehabilitation with limb movement disorders after stroke primarily emphasizes early intervention, somehow ignoring the intervention received during the recovery and the sequelae period. There is a decline in the quality-of-life of patients and aggravation of disease conditions. Therefore, improving limb motor function of stroke patients through rehabilitation is essential. Traditional rehabilitation therapy, including massage, acupuncture, physiotherapy, and electrical stimulation, has been widely employed in the clinical practice (McCrimmon et al., 2015; Yang et al., 2016; Cabanas-Valdés et al., 2021). With the progress of science and technology, several potential neurological rehabilitations are being developed using new technologies to restore movement in the stroke patients. In this review, we summarize the novel methods and applications to restore limb motor dysfunction in stroke rehabilitation, which could provide a potential therapeutic strategy against stroke in the future.

## Robot-Assisted Therapy

Robotic rehabilitation opens up a new way in stroke rehabilitation (Hesse et al., 2003; Lambercy et al., 2011; Rodgers et al., 2020). RAT uses robot equipment to treat neurological injury and assist in post-stroke rehabilitation. Therefore, it has become a research hotspot in the rehabilitation field. Based on the neuroplasticity principle and the functional remodeling mechanism, multidisciplinary therapeutics such as mechanics, rehabilitation medicine, sensing technology, and control engineering are fully integrated with providing rehabilitation training to the patients through an automated rehabilitation treatment process (Masiero et al., 2014; Morone et al., 2017, 2020). As a result, the RAT can provide high-intensity task-oriented training to stroke patients, reduce the burden on clinical staff, save medical care costs, and improve rehabilitation efficiency. For motor function recovery, robotic devices are mainly divided into exoskeleton robots and end-effector robots. The skeletal robot consists of an external electromechanical system associated with the segments and joints of stroke patients. Therefore, it can move parallel to the bones of patients and directly control individual joints, thereby reducing the abnormal posture (Chang and Kim, 2013; Calabrò et al., 2021; Moggio et al., 2021). On the other hand, end-effector robots apply mechanical forces to the distal extremities for providing force support. However, it could result in abnormal motion patterns because of the limited control over the proximal extremities.

Lamberti et al. (2021) conducted a clinical study consisting of 236 stroke patients (145 males, 91 females) admitted to a rehabilitation program with robot-assisted gait training. After accomplishing a rehabilitation program, the results have shown that the patients exhibited significant improvements in Functional Independence Measure and Functional Ambulatory Category, with a substantial recovery in women, indicating that using robotics for female stroke patients may favor a selective selection functional effect of recovery. A randomized clinical trial with 51 participants compared the effect of RAT and constraint-induced movement therapy to investigate motor recovery in stroke. Wolf Motor Function Test and Fugl-Meyer Assessment Upper Limb were designated as the primary outcome. Upper limb function and motor recovery improved in both the groups, indicating the significant potential of these new methods during stroke rehabilitation (Terranova et al., 2021). Furthermore, previous studies have observed RAT-reduced muscle tone in patients with upper limb spasms after stroke. It suggests that RAT can attenuate limb spasms caused by upper motor neuron disease, associated with repetitive motion and drafting (Veerbeek et al., 2017; Cho et al., 2018; Cho and Song, 2019). In addition, Song et al. (2021) confirmed that RAT could improve motor function and stimulate cortical activation, revealing the other mechanism involved in RAT. A multicentric, randomized controlled trial conducted in Britain compared RAT effectiveness, enhanced upper limb therapy, and usual care. A total of 770 subjects included were randomly assigned to each group. The primary outcome of successful upper limb function (Action Research Arm Test) at three months post rehabilitation did not show any advantages of RAT compared with the standard care of patients having moderate or severe upper limb dysfunction. The results indicated that RAT was not ready for routine clinical practice at the current stage (Rodgers et al., 2019).

At present, RAT has some limitations for further application. Because of the different designs and groups, there are deviations in the results of published reports. The clinical studies have significant heterogeneity in choosing different types of robotic equipment, enrolled patients, intervention time, and intervention intensity. In addition, it raises the issue of compliance among patients actively participating in the training and ensuring rehabilitation post-stroke efficiency (Yoxon et al., 2022).

## Motor Imagery Training

Motor imagery (MI), also called mental imagery, executes a particular movement or task without the actual signal output (Hanakawa, 2016; Savaki and Raos, 2019). Although the limb motor function in stroke patients is damaged, the exercise program flowchart stored in the brain is still partially preserved. Therefore, regions within the primary motor cortex, cerebellum, and basal ganglia circuits can be activated during MI (Kraft et al., 2015; Tong et al., 2017). In addition, MI can also induce the functional redistribution and regulation of neural circuits, remodeling the brain neural networks, and improving motor function relearning ability (de Vries and Mulder, 2007; Gowda et al., 2021). Furthermore, repeated training can form a normal motor reflex arc, thus, promoting limb function recovery in the stroke patients (Grabherr et al., 2015; López et al., 2019; Ladda et al., 2021). In the recent years, MIT has gradually been applied in rehabilitation as an active, low-cost, relatively simple, and efficient implementing method, attracting attention in active motor rehabilitation of stroke patients.

Motor imagery training could improve the precision and accuracy of upper limb movements and elevate the movement of hemiplegic limbs (Grabherr et al., 2015). Recently, the application of brain imaging has established the efficacy of MIT in rehabilitating stroke patients (Lioi et al., 2020; Mehler et al., 2020; Colamarino et al., 2022). Early application of MIT in post-stroke hemiplegic patients can enhance sensory information input, promote dormant synapse activation, accelerate the ischemic penumbra reperfusion, and improve cerebral blood supply, thus, enhancing the rehabilitation effect of stroke (Tavazzi et al., 2022). A recently published meta-analysis of ten randomized controlled trials showed that MIT effectively improved upper and lower limb function in stroke patients to complement traditional rehabilitation techniques (Monteiro et al., 2021). The ability to perform motor imagery involves the experience of a particular movement or task and depends on working memory, internally influencing motor representation. Therefore, MIT can improve motor performance by activating neuroplasticity in parietal lobes and related areas (Yoxon and Welsh, 2019, Yoxon and Welsh, 2020). A study applied functional task-oriented MIT in nine individuals and found that MIT could improve upper limbs and motor function of hemiplegic patients and increase visual-motor imagination ability. Page et al. conducted a series of clinical trials on MIT. They observed that compared with the control group, the MIT group (30 min of MIT twice a week for six weeks) developed the ability to perform new activities. It suggested that MI could improve the upper limb motor function and strengthen the learning ability of new skills (Page et al., 2001, 2007, 2009).

Although the efficacy of MIT in improving motor function is evident in stroke patients, many issues require clarification in future studies. First, most studies found that MIT can improve the neurological functions of stroke patients in the short term, but there are a few studies on its long-term effects in rehabilitation. In addition, published reports depicted a variable intervention time of MIT. However, the too long or too short intervention time can lead to unsatisfactory effects or make patients tired, and the choice of proper time duration and intervals are essential. At last, there is no clear standard for undergoing MIT, which may be why MIT has not been recognized and accepted by the patients. A systematic review of 32 articles observed high heterogeneity in the methodological quality and conflicting outcomes from these studies (Guerra et al., 2017). More large-scale randomized controlled trials are needed to determine the most appropriate intervention, density, duration, long-term effects of MIT, the value of MI in stroke rehabilitation, and promoting home-based rehabilitation of stroke patients.

## Virtual Reality-Assisted Therapy

Virtual reality (VR) technology uses computer synthesis of 3D environment models to create and experience virtual world technology. It is a multisource information fusion interactive 3D dynamic view. It also provides the physical behavior of the system simulation with scene display, force/tactile sensing device, position tracker, and other equipment. Immersive real feelings can be obtained through visual, auditory, or tactile real-time perception and operation of various objects inside the virtual world (Chang et al., 2020; Xiong et al., 2021). Due to high safety, high interest, timely evaluation, and feedback, VR has been gradually applied in rehabilitation treatment after stroke (Gao et al., 2021; Hao et al., 2021; Xiong et al., 2021).

A randomized controlled trial of 43 participants with stroke showed that the conventional rehabilitative approach combined with VR improves the perceived health-related quality-of-life in stroke patients (Rodríguez-Hernández et al., 2021), confirmed by other clinical studies (Erhardsson et al., 2020; Thielbar et al., 2020; Gueye et al., 2021). A systematic review of 87 studies with 3,540 participants suggested that VR interventions could effectively improve upper- and lower-limb motor function, balance, gait, and daily function of stroke patients without any cognitive benefits (Zhang et al., 2021). Moreover, the underlying mechanism may be associated with the regulation of inflammation, oxidative stress, and neuroplasticity (Huang et al., 2022). VR games have also become popular in the recent years. For example, Nintendo’s VR game comprises a wireless controller, infrared sensor, and a display screen. The sensor in the controller can alter the movement of characters in the game based on the mobility of patients to carry out various virtual games, thereby improving the upper limb movement function in the stroke patients (Hsu et al., 2011; Şimşek and Çekok, 2016; Carregosa et al., 2018; Marques-Sule et al., 2021). Some literature demonstrated no significant difference between VR and traditional training effects (Caglio et al., 2012; Crosbie et al., 2012; Laver et al., 2017). Although VR is not necessarily superior to traditional rehabilitation technology, it can be an effective alternative to rehabilitate stroke patients.

Numerous studies have identified that VR can play a beneficial role in improving limb motor function post-stroke. However, other studies with negative results suggest that more large-scale, multicentric, scientifically designed, and well-conducted clinical randomized controlled trials are needed to clarify VR effects. In addition, some VR rehabilitation equipment can only perform simple human-computer interaction. The patients need to wear complex sensing equipment, and certain virtual scenes do not provide enough immersion. Therefore, a more intelligent VR system should be designed in the future, which could better integrate motor function assessment. It should be a portable and straightforward hardware system with vivid 3D animated characters so that VR technology can improve motor dysfunction after stroke.

## Mirror Therapy

Mirror therapy also called mirror visual feedback therapy, is based on visual stimulation and flat mirror imaging. It observes the movement of healthy limbs to create the illusion of normal movement of paralyzed limbs through visual feedback, simulation of reality, and optical illusion. As a cheap, convenient, and straightforward treatment, mirror therapy cannot only for the clinical use but also for training patients at home. Mirror therapy was first proposed in 1996 and effectively reduced pain in patients with amputated arms (Ramachandran and Rogers-Ramachandran, 1996). Subsequently, studies conducted in stroke patients with hemiplegia after ictus showed that mirror therapy could significantly rehabilitate patients with upper limb motor dysfunction (Yavuzer et al., 2008; Nogueira et al., 2021; Zhuang et al., 2021). In addition, the role and impact of mirror therapy have also been explored in lower limb motor rehabilitation (Sütbeyaz et al., 2007; Li et al., 2018; Louie et al., 2019). Compared with the control group, the lower limb motor function and the daily living activities in the mirror group were significantly improved (Gandhi et al., 2020). Furthermore, a randomized controlled trial with 30 patients applied mirror therapy combined with transferable electrical stimulation to treat chronic stroke. The results depicted a significant improvement of muscle strength, Modified Ashworth Scale, Berg Balance Scale, velocity, cadence, step length, and the stride length of gait in the group treated with afferent electrical stimulation through mirror therapy (Lee and Lee, 2019).

Although the neurophysiological mechanism of mirror therapy is not fully elucidated, recent studies have revealed some possible mechanisms. Mirror therapy can reduce asymmetrical activation between the hemispheres, stimulate the ipsilateral, and the contralateral primary motor cortex, extensively activate the mirror neuron system, and induce partial motor neuron pathways on the affected side, facilitating brain function remodeling (Deconinck et al., 2015; Shih et al., 2017; Ding et al., 2019; Jaafar et al., 2021). Furthermore, the mirror neuron system can contribute to the recovery of limb motor function (Rizzolatti et al., 2009; Garrison et al., 2010). In addition, mirror therapy elevates the excitability of the cortical regions through visual feedback and promotes the remodeling of brain function, leading to motor function recovery (Calmels et al., 2006; Nojima et al., 2012).

Mirror therapy is a safe and widely used operable adjunctive therapy with a positive impact. However, the optimal intervention stage and the most effective intensity of mirror therapy have not been determined because of the varying protocols and the small sample size of the current studies. Therefore, it is suggested to standardize the implementation of mirror therapy, refine the efficacy standard, and conduct multicentric, randomized controlled trials with large sample sizes in the future. In addition, the development of more portable devices could provide updated rehabilitation services in stroke patients.

## Music Therapy

Music-based interventions have emerged as a promising tool for motor rehabilitation after stroke because they integrate motor training with multimodal stimulation (Altenmüller and Schlaug, 2015). Music therapy is currently divided into passive and active treatment based on patient participation. Passive music therapy means “listening to music,” the melody, rhythm, and other factors that act on the nervous system of the patient during listening to music. Active music therapy is the ability of a patient to imitate percussion rhythms or play musical instruments under the guidance of a music therapist (Sihvonen et al., 2017; Grau-Sánchez et al., 2020; Daniel et al., 2021). The therapeutic effect promotes the recovery of limb motor function through continuous stimulation of the motor cortex within the brain (Rojo et al., 2011; Grau-Sánchez et al., 2013; Ripollés et al., 2016). The impact of music therapy in stroke is primarily manifested through improving the exercise completion quality, strengthening the cognitive function recovery, and reducing depression and other related negative emotions after stroke (Kim et al., 2011; Le Danseur et al., 2019; Haire et al., 2021; Palumbo et al., 2021).

Music therapy can affect the brain structure and function of stroke patients and has a significant effect in treating neurological defects (Särkämö et al., 2008; Huang et al., 2021). Segura et al. (2021) designed a home-based enriched music-supported therapy program for patients recovering from chronic stroke for self-rehabilitation at home. After a ten-week intervention of three sessions per week, the patients improved the upper limb motor function by achieving most motor tests of the Minimal Detectable Change or Minimal Clinically Important Difference. Fujioka et al. (2018) investigated the effects of music-supported therapy in chronic stroke patients on motor, cognitive, and psychosocial functions compared with conventional physical training. The results revealed the beneficial effect of music therapy in all the measured aspects. Moreover, Tong et al. (2015) applied music support therapy to 30 patients with stroke. After four weeks of treatment, it was observed that the time and motion quality completing the Wolf Motor Function Test of patients are significantly better in the music group than those in the mute group. Furthermore, most patients within the music group could independently finish the playing task (Tong et al., 2015). In addition, music could act on the network structure of the brain stem, awaken the cerebral cortex, regulate the peripheral nerves, improve muscle function, and enhance the physical vitality of stroke patients (Fujioka et al., 2012b; Särkämö and Soto, 2012; Barclay et al., 2020). Music can also transmit impulses to the reticular structure of the brain stem and cerebral cortex through auditory pathways, inducing the release of brain-derived neurotrophic factors (Chen et al., 2021). Moreover, music therapy can affect the endocrine function of the hypothalamic-pituitary region through acoustic vibration. It promotes the secretion of pituitary hormones, enzymes, and active substances beneficial to nerve recovery. As a result, blood flow is regulated, and nerve cells are excited, promoting the recovery of limb motor function and improving daily living activities (Yamasaki et al., 2012).

Does the type of music affect the impact of rehabilitation? Stroke patients mainly improve their upper limb and finger function utilizing active music therapy by playing a musical instrument. However, the beneficial effects do not relate to what kind of music is being played. For passive music therapy, most studies used nostalgic music and classical music or chose the favorite music of patients (Fujioka et al., 2012a; Palumbo et al., 2021), which helped relax the patients and made them more willing to participate in the rehabilitation training. Rhythmic auditory stimulation is a neuromusic therapy technique utilizing rhythm to improve motor function. Its primary mechanism could be the synchronous effect between the auditory and motor centers, causing resonance. Fujioka et al. showed that β oscillation was related to prosodic stimulation in the auditory area, motor area, inferior frontal gyrus, and cerebellum (Fujioka et al., 2012a,^2015^). Therefore, music can cause the generation of β oscillation, which may be the better choice in rehabilitating stroke patients.

Many randomized controlled trials have established that music-based therapy can treat post-stroke motor dysfunction. However, the therapeutic effect of music therapy is still controversial (Schauer and Mauritz, 2003; Särkämö et al., 2008; Tong et al., 2015; Fotakopoulos and Kotlia, 2018). Therefore, more large randomized controlled trials and high-quality meta-analyses are needed to guide clinical practice better.

## Conclusion

**FIGURE 1:**
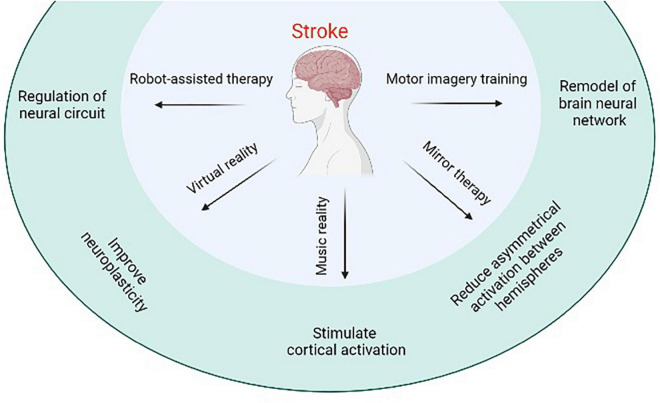
Schematic illustration of emerging the modern rehabilitation methods.

Stroke-induced neurological injury significantly reduces limb motor function in patients and leads to a decline in the quality of life. RAI, MIT, music therapy, and other emerging modern rehabilitation methods have a particular effect on improving limb motor function of stroke patients, making up for the deficiency of traditional rehabilitation measures, saving human and material resources, and becoming the hot spots of rehabilitation research (Figure 1). Several high-quality, large-sample, multicentric randomized controlled studies are needed in the future to promote positive development in stroke rehabilitation research. The current research results are neutral, and the intervention and control groups have a similar effect on movement recovery. To improve the design and implementation method of stroke rehabilitation research, we expanded the inclusion criteria to improve the inclusion rate and the universality of results, ensure the implementation of allocation hiding, and characterize the leading indicators of follow-up measures on time. The focus of future research may include but is not limited to the molecular level of the mechanism underlying rehabilitation, artificial intelligence in rehabilitation technology, and medical big data analysis, trying to achieve the best rehabilitation effect on stroke patients.

## Author Contributions

PL and FX designed and wrote the manuscript. XL provided constructive advice on the structure of this manuscript. JX, XB, JH, BZ, and FL gave constructive advice and participated in proofreading of this article. All the authors contributed to the article and approved the submitted version.

## Conflict of Interest

The authors declare that the research was conducted in the absence of any commercial or financial relationships that could be construed as a potential conflict of interest.

## Publisher’s Note

All claims expressed in this article are solely those of the authors and do not necessarily represent those of their affiliated organizations, or those of the publisher, the editors and the reviewers. Any product that may be evaluated in this article, or claim that may be made by its manufacturer, is not guaranteed or endorsed by the publisher.
